# Meeting of American Association of Obstetricians and Gynecologists

**Published:** 1896-09

**Authors:** 


### Preliminary Press Notice of the Ninth Annual Meeting of the American Association of Obstetricians and Gynecologists at Richmond, Va.

The ninth annual meeting of the American Association of Obstetricians and Gynecologists will be held at the Hotel Jefferson, Richmond, Va., Tuesday, Wednesday and Thursday, September 22, 23 and 24, 1896.

The proprietors of the "Jefferson" offer special rates to the Fellows of the Association, their families and guests, as well as to any physicians who come to attend the meeting. It is confidently expected that the railways will offer transportation at a uniform rate of a fare and a third on the certificate plan to all in attendance. Let all obtain certificates from their local ticket agents, or from the nearest point where certificates are granted.

OUTLINE PROGRAM.

The Association will meet in executive session with closed doors on Tuesday, September 22nd, at 9:30 o'clock a. m., for the election of new Fellows. The open session for the reading of papers will begin at 10 o'clock a. m. Recess for luncheon at 1 o'clock p. m. Afternoon session at 3 o'clock p. m. An evening session will be held Tuesday at 8 o'clock.

Morning session will begin Wednesday at 9:30 for the reading of scientific papers. Recess at 1 o'clock. Afternoon session at 3 o'clock. Abjournment at 5 o'clock. Executive session at 6:30 o'clock. Annual dinner at 8 o'clock p. m.

Thursday morning the session will begin at 10 o'clock. Recess at 1 o'clock. Afternoon session at 3 o'clock. Final adjournment at 5 o'clock. A full attendance is specially requested at the final session. [Program omitted.—ED.]

